# Summer Rainfall Amount Is More Important than Rainfall Frequency in Controlling the Growth and Propagation of *Leymus chinensis*, a Perennial Rhizomatous Grass in a Semiarid Ecosytem

**DOI:** 10.3390/plants15121843

**Published:** 2026-06-15

**Authors:** Zhuolin Li, Lexuan Pan, Yonggang Yi, Peilin Han, Jixiang Lin

**Affiliations:** 1 Tianhua College, Shanghai Normal University, Shanghai 201815, China; zhuolinl666@gmail.com (Z.L.); yyg2202@sthu.edu.cn (Y.Y.); 2 College of Landscape Architecture, Northeast Forestry University, Harbin 150040, China; panlexuan@nefu.edu.cn (L.P.); han-peilin@nefu.edu.cn (P.H.)

**Keywords:** rainfall amount, rainfall frequency, *L. chinensis*, vegetation growth, clonal growth

## Abstract

Climate models suggest that longer dry periods and heavier rainfall events may occur in arid and semiarid regions, which may greatly affect plant growth and propagation in these regions. Numerous studies have documented the relationship between grassland productivity and precipitation. However, the interactive effects of rainfall amount and rainfall frequency on the growth of perennial grasses with rhizomatous propagation, especially on clonal growth, have not yet been studied. In this study, the effects of three rainfall amounts and two rainfall frequencies on the vegetative traits and clonal growth traits of *Leymus chinensis*, a perennial rhizomatous species, were examined. Rainfall amount and rainfall frequency exhibited a significant interaction only for the root biomass ratio between the 0–20 cm and 20–40 cm soil layers. All traits (including height, aboveground biomass, root biomass, rhizome number, rhizome length, bud bank size, and daughter shoot number) increased markedly with increasing rainfall amount but showed little response to rainfall frequency. Only the root biomass in the 20–40 cm soil layer increased with an extended dry period between two rainfall events, resulting in a lower root biomass ratio between the 0–20 cm and 20–40 cm soil layers under the medium and high rainfall amount treatments. The size of the belowground bud bank was positively correlated with the daughter shoot number as well as the aboveground biomass, and the positive relationship between the bud bank size and daughter shoot number was strengthened with increasing rainfall amount, but was not sensitive to rainfall frequency. However, lower rainfall frequency significantly decreased the rhizome number per plant. These results highlight that summer rainfall amount is more important than rainfall frequency for the population growth of *L. chinensis* at medium and high rainfall amounts, and that lower rainfall frequency may reduce the long-term clonal growth ability of *L. chinensis* in the future. Our findings reveal the response mechanisms of *L*. *chinensis* productivity to climate change from the novel perspective of bud banks, which provides practical management insights for artificially established *L. chinensis* grasslands. This study also offers important implications for elucidating the contributions of belowground biomass production to soil carbon sequestration in grassland ecosystems.

## 1. Introduction

Human activities have caused unprecedented changes in air temperature over the past century [1], and rainfall patterns may also be altered [2,3,4]. Small rain events are advantageous for plants with shallow root systems. Larger rain events, which tend to infiltrate deeper into the soil profile where moisture persists longer, are typically necessary to initiate plant growth and production [5,6]. Wang et al. showed that the drought durations in summer and autumn were longer than those in spring and winter in Northeastern China [4]. This may strongly affect arid and semiarid ecosystems [7,8], as productivity in these regions is largely limited by water availability, especially in summer.

Currently, increases in the frequency and magnitude of heavy and extreme rainfall events have been reported in many regions [9,10]. Most research suggests that mean annual precipitation is positively related to aboveground net primary productivity (ANPP) of plants, and these findings are supported by studies of 74 samples from north temperate grasslands and steppes dominated by *Stipa capillata* in Inner Mongolia, China [11]. However, some contrasting results have also been reported [12]. In addition, many phenomena cannot be explained by precipitation amount alone. Swemmer et al. suggested that rainfall distribution is determined by three factors: the average single rainfall amount during the growing season, rainfall frequency, and the average interval between two rainfall events, each of which may have different effects among grasslands with different water conditions [13]. The effects of the average single rainfall amount or rainfall frequency on ANPP may be more important than total precipitation in semiarid grasslands. Based on these findings, many studies have been conducted to explore whether rainfall amount or rainfall frequency is more important in different ecosystems. Under fewer but larger rainfall events, productivity increases are more common in dry ecosystems (46% positive; 20% negative), whereas responses are typically negative in wet ecosystems (28% positive; 51% negative) [10]. Research methods have mainly focused on short-term artificial experiments or long-term regression analyses of ecological data at local scales or along natural rainfall gradients [14,15,16,17]. Numerous studies have documented the relationship between productivity and precipitation, but most focus on aboveground production (ANPP), while the effects on belowground production (BNPP) remain poorly understood [16,17,18]. Because BNPP is estimated to account for 33–78% of total NPP, BNPP is crucial in arid and semiarid regions [17]. A few studies showed that responses of ANPP and BNPP decoupled in many ecosystems [19,20]. Furthermore, BNPP and precipitation were uncorrelated [21].

Most grassland plants are perennial clonal species, and clonality is an important characteristic of plant bodies [22,23]. In perennial-dominated grassland ecosystems, aboveground vegetation regenerates annually. Therefore, the loss of aboveground structures may be less important for plant survival, growth, and reproduction than belowground clonal organs, as most aboveground regeneration originates from the belowground bud bank. Consequently, clonal growth is more important than individual carbon absorption in clonal plants [24]. The importance of bud banks has now been documented in numerous habitats [25,26]. Many studies have demonstrated positive relationships between bud bank size and factors such as precipitation, summer nocturnal warming, and nitrogen deposition [26]. However, responses of clonal growth to precipitation patterns remain largely neglected due to the difficulty of belowground sampling.

In this study, *L. chinensis* was selected as the study species because it is a typical perennial C_3_ grass with rhizomatous propagation and is a dominant species in the Songnen Grassland of Northeastern China, a typical semiarid grassland ecosystem. It is also an important forage resource due to its high contents of protein, minerals, and carbohydrates, as well as its good palatability to many large herbivores. Moreover, water availability is one of the most important limiting factors in the Songnen Grassland. Therefore, three rainfall amounts and two rainfall frequencies were simulated based on the historical variations in rainfall data. According to previous studies, the following hypotheses are proposed: (1) rainfall amount is more important than rainfall frequency in controlling the growth of *L. chinensis*; (2) low rainfall frequency reduces vegetative traits and clonal growth traits; and (3) changes in rainfall pattern alter the relationship between the size of the belowground bud bank and the aboveground daughter shoot number.

## 2. Materials and Methods

### 2.1. Study Area Profiles

The *L. chinensis* used in this study was grown in the field station of the Institute of Grassland Science, Jilin Province, in Northeastern China (123°44′ E, 44°44′ N, 167 m elevation). This region has a semiarid continental monsoon climate with a frost-free period of approximately 140 days. The annual mean temperature is 6.4 °C, and the annual mean precipitation is 361.6 mm, more than 70% of which occurs during the summer months from June to August. The main soil type in this ecosystem is saline–alkaline Mollisol.

### 2.2. Plant Culture

Seeds of *L. chinensis,* collected from an artificial grassland at the Songnen Field Station, were sown in the pot at Northeast Normal University at the end of July, when precipitation is abundant and temperatures are suitable for seedling establishment. The soil was sieved through a 2 mm mesh to remove roots and other visible debris and thoroughly mixed. Soil organic matter content, total nitrogen, and pH were 0.3%, 6.8%, and 8.63, respectively. The pots (diameter of 23.2 cm and height of 21.3 cm) were then placed outdoors to receive normal sunlight, with only routine watering provided during this period. By the end of October, the pots were buried in the soil, ensuring the newly grown *L. chinensis* could survive the winter successfully.

This experiment was initiated in early May of the second experimental year. The entire experiment was conducted in an open-top greenhouse with bilateral ventilation to ensure near-natural growing conditions. The greenhouse roof was covered overnight and dynamically adjusted (opened or closed) during the daytime according to real-time weather conditions, thereby minimizing the artificial interference of the greenhouse facility on the internal micro-environment.

Experimental plots were prepared by excavating pits with dimensions of 150 cm in length, 200 cm in width, and 40 cm in depth. Tall polyvinyl chloride (PVC) cylinders with an inner diameter of 25 cm and a height of 40 cm were adopted as cultivation containers. These cylinders were designed with open bottoms to establish a bucket-free cultivation system, eliminating the growth restriction of traditional bucket bases on the downward extension of plant rhizomes. The cylinder height of 40 cm was determined based on preliminary experimental results, which confirmed that this depth could effectively prevent bottom leakage under maximum rainfall conditions.

**Figure 1:**
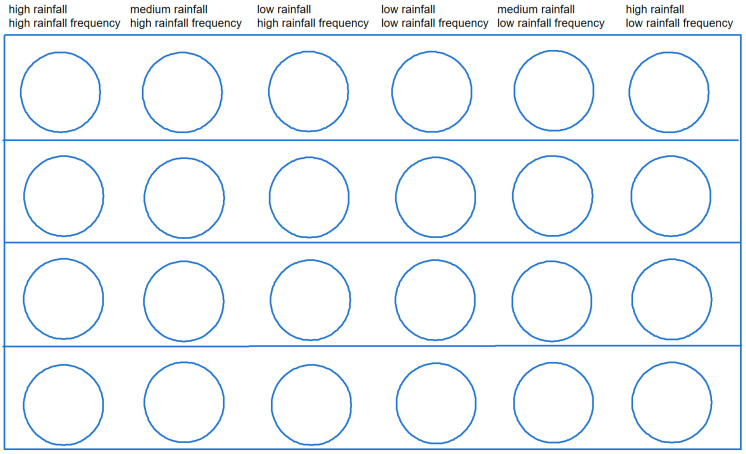
Three precipitation gradients and two rainfall frequency treatments.

All PVC cylinders were arranged in a 4 × 6 grid pattern within the excavated pits (Figure 1). The gaps between cylinders and pit walls were backfilled and compacted with field soil to stabilize the cylinders and simulate the natural growth environment of *Limonium chinense*. Each cylinder was then filled with sieved and homogenized meadow saline–alkali soil (the native soil for *L. chinense* growth), with the soil level inside and outside the cylinders kept consistent and approximately 1 cm below the cylinder top.

Overwintered *L. chinense* seedlings were subsequently transplanted into the PVC cylinders. Initially, fifteen seedlings with newly developed rhizomes and no connected ramets were planted in each cylinder. One week after transplantation, ten seedlings of uniform growth status and size were retained per cylinder, with the extra seedlings removed. To guarantee successful seedling establishment, all experimental plants were irrigated uniformly once every three days throughout the seedling acclimation stage.

### 2.3. Rainfall Manipulation

The water control experiment was initiated on June 1 and lasted for three months. The experiment consisted of three rainfall gradient levels and two rainfall interval treatments, forming six experimental treatments in total, with four biological replicates for each treatment. The experimental precipitation regime was designed based on multi-year meteorological data of the Songnen Grassland, where the average precipitation in June, July, and August is 72 mm, 78 mm, and 76.5 mm, respectively. Specifically, the summer precipitation in the wettest local year was approximately 42% higher than the multi-year average, while that in the driest year was 38% lower than the average. Accordingly, three total rainfall levels were established in this experiment, including ambient precipitation, 40% increased precipitation, and 40% decreased precipitation (Table 1).

Two rainfall frequency regimes were set for the experimental period. The high-frequency treatment adopted the average summer rainfall frequency of the local area, with 12 rainfall events in June, 12 events in July, and 9 events in August. The low-frequency treatment was set to one-third of the average summer rainfall frequency, with 4 rainfall events in June, 4 events in July, and 3 events in August (Table 2 and Table 3).

### 2.4. Plant Harvest

At the end of the experiment (8 September), all plants were harvested. Each plant in every PVC cylinder was carefully separated from the soil and labeled for examination. Roots from the 0–20 cm and 20–40 cm soil layers were collected separately. The total number of rhizomes, belowground buds longer than 1 mm, and corresponding daughter shoots per parent shoot were counted immediately. The total rhizome length for each parent shoot was also measured. Bud classification was carried out with reference to Li et al. [27]. Parent shoots, daughter shoots, roots from the 0–20 cm and 20–40 cm soil layers, and rhizomes were separated and weighed after drying at 65 °C for 72 h.

### 2.5. Data Analyses

Two-way analyses of variance were carried out to compare the effects of rainfall amount and frequency and their interaction on growth traits (height, biomass) and clonal growth (rhizome number, rhizome length, buds, daughter shoots). The ratio between 0 and 20 cm root and 20–40 cm root biomass was log10–transformed before analyses to keep homogeneity.

## 3. Results

### 3.1. Vegetative Traits

Rainfall amount treatments induced significant variations in plant vegetative traits. In contrast, rainfall frequency only exerted significant effects on aboveground biomass and root biomass in the 20–40 cm soil layer. Additionally, a significant interactive effect between rainfall amount and rainfall frequency was observed (Table 4), whereby the influence of rainfall frequency on the root biomass ratio between the 0–20 cm and 20–40 cm soil layers was dependent on rainfall amount.

**Figure 2:**
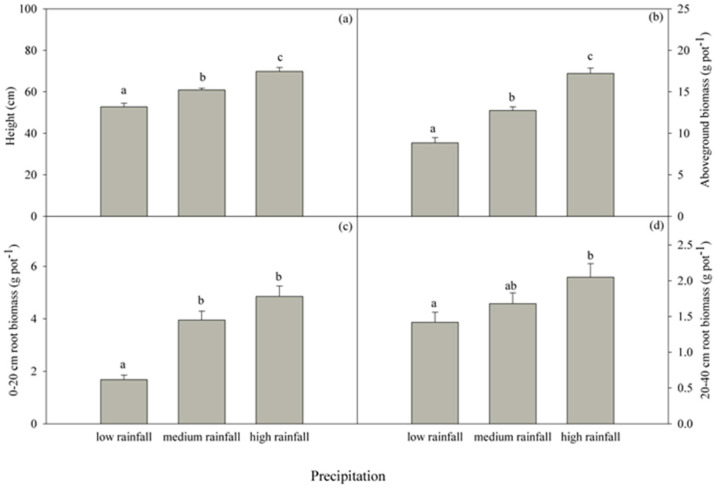
Effects of precipitation amount on vegetative traits of *Leymus chinensis*. (**a**) Plant height (cm); (**b**) aboveground biomass (g pot^−1^); (**c**) 0–20 cm root biomass (g pot^−1^); (**d**) 20–40 cm root biomass (g pot^−1^). Different lowercase letters indicate significant differences among precipitation treatments at *p* < 0.05.

With the increase in rainfall amount, plant height and aboveground biomass both increased significantly (Figure 2a,b). For the 0–20 cm soil layer, low rainfall treatment significantly reduced root biomass by 57.5%, while no significant difference was detected between the medium and high rainfall treatments (Figure 2c). In the 20–40 cm soil layer, root biomass under high rainfall conditions was markedly higher than that under low rainfall conditions (Figure 2d).

**Figure 3:**
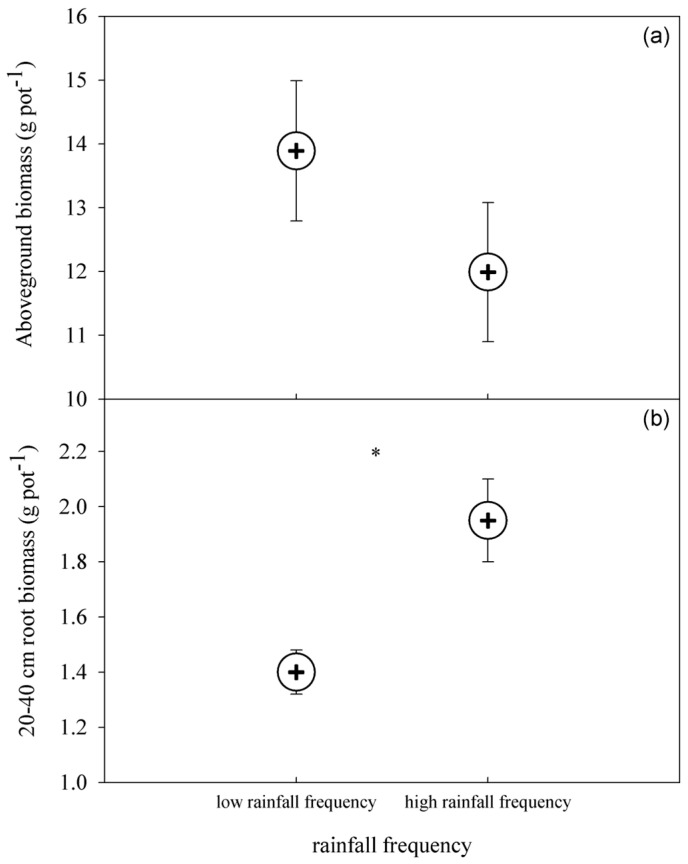
Effects of rainfall frequency on aboveground biomass (**a**) and root biomass (**b**) in the 20–40 cm soil layer of *Leymus chinensis*. Asterisks indicate significant differences between the two rainfall frequency treatments.

Plants under high rainfall frequency exhibited higher aboveground biomass relative to those under low rainfall frequency, though the difference was not statistically significant (Figure 3a). Rainfall frequency significantly affected root biomass in the 20–40 cm soil layer, with low-frequency rainfall conditions contributing to greater root biomass compared with high-frequency conditions (Figure 3b).

**Figure 4:**
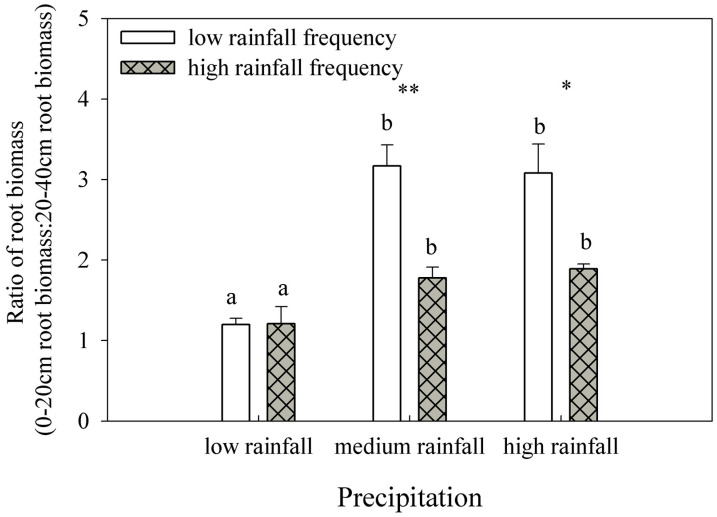
Effects of precipitation amount and frequency on the ratio of root biomass in the 0–20 cm layer to that in the 0–40 cm layer of *Leymus chinensis*. Identical lowercase letters indicate no significant differences among precipitation levels under the same frequency, while asterisks denote significant differences between the two rainfall frequencies at the same precipitation level.

Under both rainfall frequency regimes, the root biomass ratio of the 0–20 cm to the 20–40 cm soil layer declined under low rainfall conditions. Specifically, under medium and high rainfall levels, low rainfall frequency reduced the root biomass ratio by 43.8% and 38.6%, respectively, whereas no pronounced variation in this ratio was observed under low rainfall conditions (Figure 4). These findings demonstrated that plants tended to develop deeper root systems under the combined conditions of low rainfall amount and low rainfall frequency.

### 3.2. Clonal Growth Traits

Rainfall amount treatments caused significant differences in all clonal growth traits, whereas rainfall frequency treatments significantly affected the rhizome number and rhizome length (Table 4).

Almost all clonal growth traits increased on average with increasing rainfall amount, indicating that higher precipitation promoted the clonal propagation of *L. chinensis.* Only the bud number under medium and high rainfall treatments shows no significant difference (Table 5).

**Figure 5:**
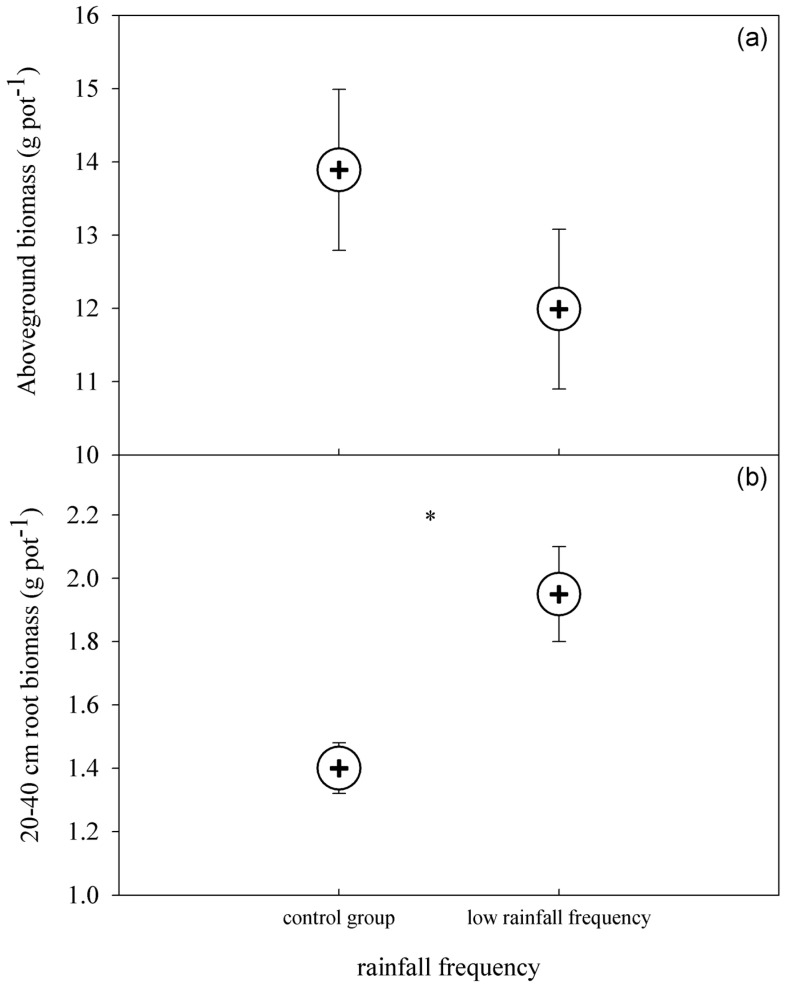
Effects of rainfall frequency on rhizome number and rhizome length of *Leymus chinensis*. Asterisks indicate significant differences between the two rainfall frequency treatments. (**a**) aboveground biomass (**b**) root biomass.

Plants under the high rainfall frequency treatment (Control group) produced significantly more rhizomes than those under the low rainfall frequency treatment (*p* < 0.05, Figure 5a). Plants under high rainfall frequency treatment (Control group) also showed, on average, 26% greater rhizome length than those under low rainfall frequency treatment (Figure 5b).

### 3.3. Relationships of Belowground Bud Bank with Daughter Shoot Number and Aboveground Biomass Under Different Rainfall Treatments

**Figure 6:**
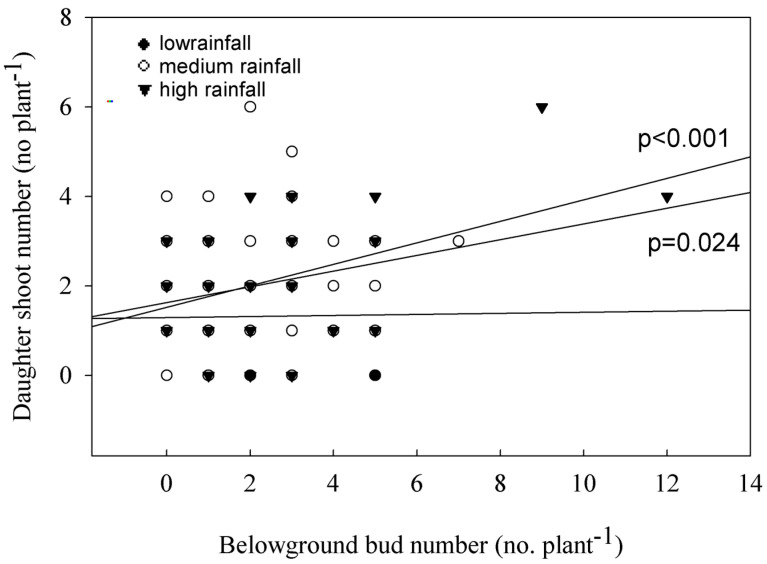
Relationships between belowground bud number and daughter shoot number across three precipitation levels.

The size of the belowground bud bank exhibited a significant positive correlation with daughter shoot number under the medium rainfall and high rainfall treatments, whereas no significant relationship was observed under the low rainfall treatment (Figure 6). Moreover, the relationship between the bud bank size and daughter shoot number was strengthened with increasing rainfall amount.

**Figure 7:**
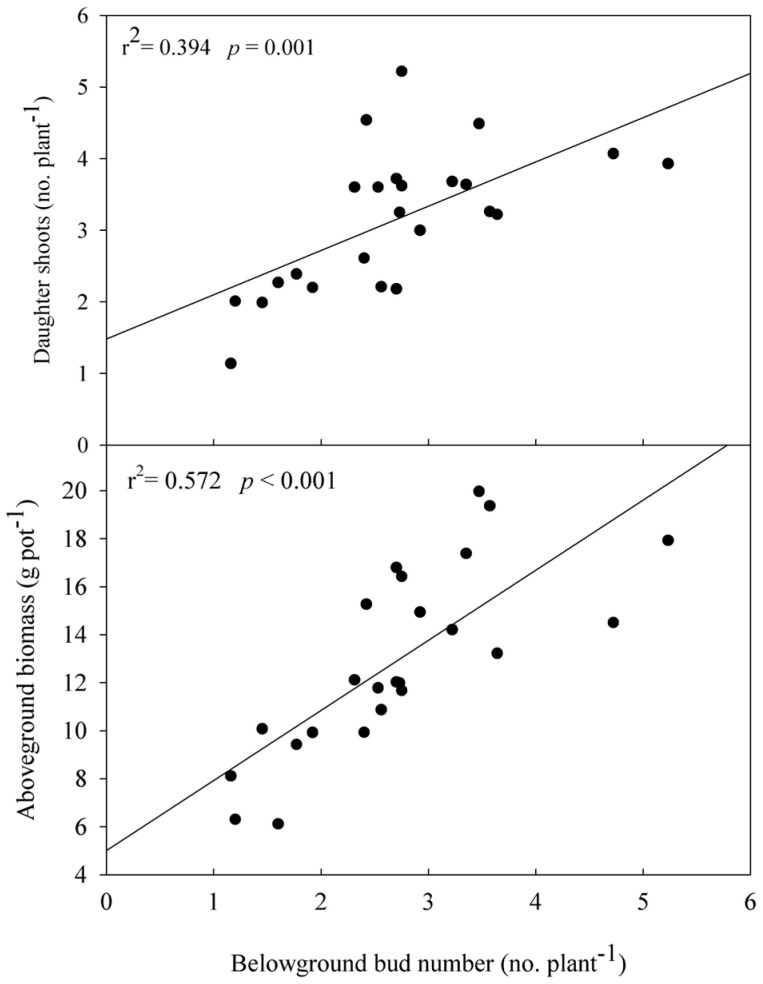
Relationships of belowground bud number with daughter shoot number and aboveground biomass.

In addition, the size of the belowground bud bank was significantly correlated with both daughter shoot number and aboveground biomass, indicating that the bud bank is a major contributor to aboveground biomass (Figure 7).

## 4. Discussion

Drought and its impact on terrestrial ecosystems are arguably the most studied climate extreme [28]. Within-season precipitation regimes are also predicted to intensify in the future, characterized by larger rainfall events and longer dry periods [10,29]. Drought stress induced by summer evaporation has intensified; however, plant growth is most vigorous during summer. This means that extreme rainfall events in summer may affect plants more severely. Songnen Grassland belongs to a semiarid region, dominated by the *L. chinensis* population. *L. chinensis* is an important forage. According to previous studies, its productivity is mainly determined by precipitation, and production is contributed by clonal growth. Therefore, we want to know how productivity and clonal growth of *L. chinensis* respond to changes in rainfall events, including larger individual rainfall events and extended dry periods between events, which one is more crucial? The results of this study support Hypotheses 1 and 3, but only partially support Hypothesis 2, as low rainfall frequency was not found to significantly decrease the size of the belowground bud bank or aboveground daughter shoot number. These results suggest that the summer rainfall amount may be more crucial than rainfall frequency. Reduced rainfall frequency does not significantly affect *L. chinensis* under medium or high rainfall conditions, but imposes significant negative effects in drier years.

Whether rainfall amount or rainfall frequency is more important in controlling plant individuals, population dynamics, and community composition remains controversial [10,16]. Some studies suggest that increased within-season precipitation variability reduces ANPP in mesic systems and increases or has no effect on ANPP at low MAP [21,30]. In a controlled experiment on an annual species, Gao et al. showed that both vegetative and reproductive traits were more strongly explained by rainfall amount than by rainfall frequency [31], and similar conclusions have been reported in other studies [32]. In our study, all traits showed significant differences under the treatment of rainfall amount; however, only a few traits, including the root biomass in the 20–40 cm soil layer, the root biomass ratio between the 0–20 cm and 20–40 cm soil layers, and rhizome number, responded significantly to rainfall frequency (Table 4). These results suggest that summer rainfall amount may be more important than rainfall frequency in controlling the growth of *L. chinensis,* especially in dry conditions.

We found that root biomass was highest in the treatment that received small-frequent rain events (medium rainfall group), and lowest in low rainfall and low rainfall frequency. Our results add to a growing body of work showing the positive effects of small rain events on production [17,33]. A large rainfall event did not increase belowground biomass. Collins and Brown also found that small, frequent rain events promoted comparable rates of ANPP to large, infrequent rain events in the absence of nitrogen enrichment in the dryland region [17]. So, in arid and semiarid regions, although small rain events often do not percolate very deeply, persistent moisture availability in the top few centimeters of soil where roots are abundant can maintain plant production in this system where most rain events are small [17]. Therefore, small rain events may have greater ecological significance for maintaining grass productivity in arid and semiarid regions. Additionally, it was discovered that the ratio of root biomass at 0–20 cm to that at 20–40 cm was significantly diminished, regardless of rainfall frequency (Figure 4). This suggests that the water shortage or strengthening of transpiration resulted in a reduction in the water content in the upper soil layer. The roots of *L. chinensis* were more distributed towards the deep soil to acquire a greater amount of soil moisture. This response demonstrates strong plasticity to rainfall variation and may enhance fitness under unpredictable environments in semiarid regions. These findings are consistent with studies conducted in prairie ecosystems [29,34,35], but partly different from Zhang et al. This discrepancy can be attributed to divergent precipitation addition patterns. Zhang et al. augmented the magnitude of each natural precipitation event across the entire growing season, which generated numerous low-intensity rainfall pulses [36]. In contrast, our study kept the total precipitation volume consistent across different rainfall frequency treatments. Intense rainfall facilitates deeper soil water infiltration, thereby promoting the growth of deep roots to utilize subsurface soil water. By comparison, light rainfall is mostly retained in topsoil layers and favors the development of shallow roots [37].

Aboveground biomass was positively correlated with increasing rainfall, but root biomass was not. This suggested that the ratio of aboveground net primary productivity (ANPP) to belowground net primary productivity (BNPP) of *L. chinensis* was uncorrelated under the conditions of medium rainfall and high rainfall, which is consistent with the prior findings regarding the non-coupling of underground and aboveground biomass [17,19,20]. Sun et al. showed that aboveground production is driven more by the environment [38], whereas belowground production is more a function of vegetation and soil properties, factors that likely contribute to a weak correlation between these important carbon cycle processes. The change in aboveground biomass may have a lag effect after belowground biomass. Deeper infiltration also results in reduced evaporative loss, potentially resulting in more available water for plant growth and total C uptake [39]. Importantly, deep root production might contribute to increased long-term soil C sequestration.

The bud bank represents the most crucial source of the aboveground net primary productivity (ANPP) of *L. chinensis*, and the regeneration of *L. chinensis* primarily relies on the bud bank. In this research, it was discovered that the number and length of rhizomes increase as the rainfall amount rises. Clonal plants have two fundamental strategies to cope with disturbance: tolerance and avoidance. Under disturbance, clonal plants may reduce resprouting from the belowground bud bank while increasing dispersal through seeds [23]. In the present study, the positive correlation between underground bud bank density and aboveground daughter shoot number intensified with increasing rainfall, indicating that precipitation promotes bud emergence (Figure 6). The interesting finding was that the total number of buds did not show a significant difference between the medium rainfall and high rainfall treatments, yet the number of daughter shoots did (Table 5). This also suggested that a high rainfall amount stimulates the transformation of buds into daughter shoots, which contributes to the aboveground biomass (Figure 7). Conversely, drought—a form of disturbance—impairs bud-to-progeny output due to meristem constraints [24]. In addition, lower rainfall frequency was found to significantly decrease rhizome number and slightly reduce rhizome length. This response primarily stemmed from the effect of lower rainfall frequency and low rainfall amount. This indicates that small, frequent events with low rainfall would lead to severe drought conditions. Rhizomes, as key vegetative reproductive organs, may reduce their abundance under drought conditions. Instead of ceasing growth, they deploy exploratory growth to colonize alternative habitats, where favorable humidity can promote bud germination and daughter shoot emergence. Rhizome also potentially influences the size of the belowground bud bank. Studies have shown a significant positive correlation between the biomass of living rhizomes and the density of the bud bank, indicating that rhizome biomass can reflect bud bank density to some extent [40]. Therefore, although rainfall frequency did not affect the bud bank significantly in this study, a reduction in asexual reproductive organs may subsequently impair reproductive capacity, thereby impacting population renewal and productivity. Unfortunately, very few studies have examined the effects of rainfall frequency on clonal growth. The present study provides novel evidence from the perspective of bud bank dynamics.

In conclusion, the present study showed that both vegetative traits and clonal growth traits of *L. chinensis* depend more strongly on summer rainfall amount than on rainfall frequency. Therefore, rainfall amount remains the key factor regulating *L. chinensis* populations when plants can tolerate extended dry periods between rainfall events. However, lower rainfall frequency will aggravate the negative effects of drought. So decreasing rainfall frequency may weaken the long-term clonal growth ability of *L. chinensis*.

Overall, our findings reveal the response mechanisms of *L*. *chinensis* productivity to climate change from the novel perspective of bud banks, which provides practical management insights for artificially established *L. chinensis* grasslands. This study also offers important implications for elucidating the contributions of belowground biomass production to soil carbon sequestration in grassland ecosystems.

## Figures and Tables

**Table 1:** Total rainfall volume and event frequency across experimental treatments. Values in bold represent the mean precipitation amount recorded at this site over the past 12 years.

Month	Precipitation Level	Total Precipitation (mm)	Frequency Level	Number of Rainfall Events	Single Rainfall Amount (mm)
June	Low	42	Control	12	3.5
June	Low	42	Low	4	10.5
June	Medium	**72**	Control	12	6.0
June	Medium	**72**	Low	4	18.0
June	High	102	Control	12	8.5
June	High	102	Low	4	25.5
July	Low	47	Control	12	4.0
July	Low	47	Low	4	12.0
July	Medium	**78**	Control	12	6.5
July	Medium	**78**	Low	4	19.5
July	High	109	Control	12	9.0
July	High	109	Low	4	27.0
August	Low	46	Control	9	5.0
August	Low	46	Low	3	15.0
August	Medium	**76.5**	Control	9	8.5
August	Medium	**76.5**	Low	3	25.5
August	High	107	Control	9	12.0
August	High	107	Low	3	36.0

**Table 2:** The average rainfall frequency each month in summer (June–August).

Rainfall Frequency (Times)	Month		
	June	July	August
Control	12	12	9
Low	4	4	3

**Table 3:** Watering schedules in June, July and August. Bold fonts represent high-frequency watering dates, and red bold fonts denote low-frequency watering dates.

1	2	**3**	4	**5**	6	7
**8**	9	**10**	11	12	**13**	14
**15**	16	17	**18**	19	**20**	21
22	**23**	24	**25**	26	27	**28**
29	**30**	(June)				
1	2	**3**	4	**5**	6	7
**8**	9	**10**	11	12	**13**	14
**15**	16	17	**18**	19	**20**	21
22	**23**	24	**25**	26	27	**28**
29	**30**	31	(July)			
1	2	**3**	4	5	6	**7**
8	9	**10**	11	12	13	**14**
15	16	**17**	18	19	20	**21**
22	23	**24**	25	26	27	**28**
29	30	**31**	(August)			

**Table 4:** Results of two-way ANOVA examining the effects of precipitation amount and frequency on traits of *Leymus chinensis*. The values in the table are F-values.

	Rainfall Amount	Rainfall Frequency	Rainfall Amount × Rainfall Frequency
Vegetative traits			
Height	27.411 ***	1.205	0.291
Aboveground biomass	76.550 ***	11.893 **	0.972
0–20 cm root biomass	31.222 ***	2.708	0.192
20–40 cm root biomass	4.714 *	7.465 *	0.305
Ratio of root biomass (0–20 cm:20–40 cm)	32.573 ***	18.833 ***	4.267 *
Clonal growth			
Rhizome number	55.984 ***	28.957 ***	0.125
Rhizome length	41.898 ***	5.13 *	1.745
Total belowground buds	12.931 ***	1.47	0.361
Total daughter shoots	29.006 ***	2.575	0.07

(* indicates *p* < 0.05, ** indicates *p* < 0.01, *** indicates *p* < 0.001).

**Table 5:** Effects of precipitation amount on clonal growth traits of *Leymus chinensis*. Different lowercase letters indicate significant differences among precipitation treatments (*p* < 0.05).

Precipitation	Rhizome Number	Rhizome Length	Total Buds	Total Daughter Shoots
Low rainfall	2.52 ± 0.15 a	16.10 ± 1.48 a	1.76 ± 0.18 a	2.10 ± 0.15 a
Medium rainfall	3.32 ± 0.17 b	32.59 ± 2.70 b	2.85 ± 0.15 b	3.27 ± 0.18 b
High rainfall	4.02 ± 0.12 c	55.57 ± 5.12 c	3.53 ± 0.35 b	4.11 ± 0.22 c

## Data Availability

The original contributions presented in this study are included in the article. Further inquiries can be directed to the corresponding author.
